# Translation, Cultural Adaptation, and Validation of the International Patient Decision Aid Standards Minimal Criteria Instrument for the Portuguese Population

**DOI:** 10.1177/23814683251386451

**Published:** 2025-10-18

**Authors:** Micaela Gregório, Andreia Teixeira, Mariana Teixeira, Inês Marques, Ana Sofia Correia, Phillippa May Bennett, Helen Carter, Dawn Stacey, Carlos Martins

**Affiliations:** RISE-Health, Faculty of Medicine, University of Porto, Alameda Prof. Hernâni Monteiro, Porto, Portugal; #H4A Primary Healthcare Research Network, Guifões, Portugal; Rede de Investigação nos Cuidados de Saúde Primários do Alto Ave, ULS Alto Ave, Guimarães, Portugal; Department of Community Medicine, Information and Health Decision Sciences (MEDCIDS), Faculty of Medicine, University of Porto, Alameda Prof. Hernâni Monteiro, Porto, Portugal; RISE-Health, Faculty of Medicine, University of Porto, Alameda Prof. Hernâni Monteiro, Porto, Portugal; #H4A Primary Healthcare Research Network, Guifões, Portugal; Department of Community Medicine, Information and Health Decision Sciences (MEDCIDS), Faculty of Medicine, University of Porto, Alameda Prof. Hernâni Monteiro, Porto, Portugal; ADiT-LAB, Instituto Politécnico de Viana do Castelo, Rua Escola Industrial e Comercial Nun’Álvares, Viana do Castelo, Portugal; Medical translator, Vila do Conde, Portugal; EN-PT translator & translation project manager, Madrid, Spain; Medical translator and writer, Lousã, Portugal; Faculty of Arts and Humanities, University of Coimbra, Coimbra, Portugal; Centre for English, Translation, and Anglo-Portuguese Studies, New University of Lisbon, Portugal; Translator EN<>PT, Tomar, Portugal; School of Nursing, University of Ottawa, Ottawa, Canada; Centre for Practice Changing Research, Ottawa Hospital Research Institute, Ottawa, Canada; RISE-Health, Faculty of Medicine, University of Porto, Alameda Prof. Hernâni Monteiro, Porto, Portugal; #H4A Primary Healthcare Research Network, Guifões, Portugal

**Keywords:** decision making, decision support techniques, decision aids, translation, cultural adaptation, validation studies, IPDAS minimal criteria

## Abstract

**Introduction.** Patient decision aids support health care decision making by dynamically integrating evidence-based information with patients’ values, goals, and preferences. However, most of these aids are available only in English, limiting accessibility for non–English-speaking populations. Although Portuguese is one of the most spoken languages worldwide, validated decision aids and evaluation instruments in Portuguese remain scarce. **Objectives.** To translate, culturally adapt, and conduct preliminary content validation of the International Patient Decision Aid Standards (IPDAS) Minimal Criteria instrument for the Portuguese population. **Methods.** A multidisciplinary team conducted a structured linguistic validation process, including forward and backward translation, synthesis meetings, expert committee review, and pilot testing. Clarity and content relevance were evaluated by a panel of 10 experts using a dichotomous clarity scale and a 4-point relevance scale. Quantitative measures included percentage agreement, Fleiss’ kappa, and item-level and scale-level content validity indices (I-CVI, S-CVI/Ave). Qualitative input was gathered through open-ended responses and discussion during the expert panel meeting. **Results.** Five of the 44 criteria were identified as unclear by more than 20% of the expert panel and were revised. The overall agreement was high (0.93 [0.89; 0.97]), but Fleiss’ kappa indicated low interrater agreement (0.03 [−0.01; 0.08]). Eleven criteria were rated by at least 1 panel member as less relevant or in need of revision and were refined accordingly. For all criteria, the I-CVI was >0.79, and the overall S-CVI/Ave was 0.97. Fleiss’ kappa for content validity was −0.02 [−0.06; 0.02]. **Conclusions.** The Portuguese version of the IPDAS Minimal Criteria demonstrated strong content validity and linguistic appropriateness. This adapted instrument will enable more rigorous evaluation of patient decision aids in Portuguese-speaking contexts and support broader implementation of shared decision making.

**Highlights:**

- This study provides the first content-validated version of the IPDAS Minimal Criteria in Portuguese, addressing a significant gap in shared decision-making tools.
- The instrument supports Portuguese-speaking researchers and clinicians in systematically assessing decision aids for quality and usability.
- These findings highlight the importance of cultural adaptation in ensuring the applicability and effectiveness of decision support tools across different populations.
- This study advances the field of decision-making research by fostering equitable access to high-quality decision aids in clinical practice.

In the past, physicians have held a dominant role in health care decisions, particularly in contexts involving medical interventions such as treatments, screening, or procedures. Historically, their recommendations were guided by clinical expertise and the assumption that they knew what was best for their patients. This approach is known as the “paternalistic model,” which is grounded in Parsons’ conceptualization of the sick role.^1^ Under this model, physicians make decisions based on what they believe is in the patient’s best interest without necessarily considering the patient’s preferences.

However, over recent decades, there has been a paradigm shift toward more collaborative models of care. Shared decision making is a dynamic and collaborative process in which clinicians and patients work together to make health care decisions, integrating the best available clinical evidence with the patient’s own values, goals, and preferences.^2^ This approach recognizes patients as active agents in their care, promoting not only their autonomy but also their engagement in decisions that respect their health.^3,4^ This consultation model is especially important when more than 1 medically reasonable option exists and when no single option is clearly superior (i.e., in situations of clinical equipoise). In such cases, choosing among alternatives requires not only clinical reasoning but also an understanding of what matters most to the patient.

To support shared decision making, interventions known as patient decision aids (PDAs) were designed. These tools provide evidence-based information about the risks and benefits of different options and help patients understand their options, anticipate potential outcomes, and clarify what matters most to them.^5,6^ Evidence from a systematic review of 209 randomized controlled trials demonstrates that patients exposed to decision aids feel more informed, better understand the benefits and harms of their options, and are more likely to engage in decision making.^7,8^ While these interventions are not essential for shared decision making, they can help make the process easier and ensure that the patient remains at the center of the decision-making process.

However, despite their benefits, most of these instruments are developed in English, limiting access for patients in other linguistic and cultural settings, including Portuguese-speaking populations.

A recent Portuguese study revealed that many family doctors confuse PDAs with clinical decision support tools.^9^ While both aim to improve care quality, PDAs are focused on informing and empowering patients in preference-sensitive decisions, whereas clinical decision support tools assist health care professionals in applying clinical guidelines or algorithms to optimize medical decisions. Despite some confusion between these concepts, the integration of PDAs in primary health care was received positively, with barriers to implementation including insufficient funding, time constraints, and the absence of validated Portuguese-language translations.^9^

Concerns about the quality and consistency of PDAs first arose in 2003 when evidence showed how they could influence patient choices.^10^ The International Patient Decision Aid Standards (IPDAS) Collaboration, an international and multidisciplinary group, developed a set of criteria to assess the quality of PDAs, which has since been refined and used globally.^6,10–13^ Among these, the IPDAS Minimal Criteria (v4.0, 2013) identify the essential elements a PDA must include to support high-quality decision making. Although versions of these criteria exist in other languages, such as Spanish, Chinese, and Japanese, no validated version exists in Portuguese.

This study aimed to translate, culturally adapt, and assess the content validity of the IPDAS Minimal Criteria instrument (v4.0, 2013) for the Portuguese population, addressing a significant gap and providing a validated tool to support the evaluation and development of decision aids in Portuguese-speaking health care settings.

## Methods

### Study Design

The translation, cultural adaptation, and content validation of the IPDAS Minimal Criteria v4.0 (2013) for the Portuguese population were conducted based on the methodology of Sousa and Rojjanasrirat.^14^ Cultural adaptation is more than a linguistic process; it ensures that translated instruments maintain conceptual, semantic, and content equivalence within the cultural context of the target population.^14–17^

### Translation, Cultural Adaptation, and Content Validation of the Instrument

Before beginning the translation process, the authors sought and received permission for the translation and adaptation of the instrument from the IPDAS Steering Committee, which granted this authorization via e-mail.

Once authorization had been granted, some changes were proposed to the original version of the instrument, including the addition of a new qualifying criterion stating the target audience of the decision aid, as suggested by Martin et al.^18^ Furthermore, for the qualifying criterion, “The PDA describes the features of options to help patients imagine what it is like to experience their physical, emotional, and social effects,” we were informed that the committee allows the use of an explicit values clarification exercise as an alternative to this item. This adjustment acknowledges that decision aids meeting this description would be longer compared with those using a values clarification exercise. Therefore, the research team decided to include this alternative in the criteria. In addition, the committee recommended using a dichotomous scale to evaluate certification and quality criteria. According to the IPDAS Steering Committee co-lead, most people find the 4-point response scale difficult to follow without training and instead prefer a yes/no response. Following these amendments, we obtained permission to use the modified version of the original IPDAS instrument in the linguistic validation process. The modified version of the criteria translated for this study is provided in Appendix 1.

The research team decided to use the methodology of Sousa and Rojjanasrirat^14^ since it had been suggested when permission to translate the instrument was granted. Table 1 summarizes the recommended 7-step cross-cultural adaptation and validation process. Steps 1 to 5 encompass the translation and cultural adaptation of the instrument, whereas steps 6 and 7 focus on validating the translated instrument. In this initial study, we followed steps 1 to 5 to create a translated, culturally adapted, and content validated instrument in Portuguese. Steps 6 and 7 will be addressed in future psychometric validation studies.

**Table 1:** Summary of the Translation, Cultural Adaptation, and Content Validation Process of the Portuguese IPDAS Minimal Criteria Instrument

Step	Stage of Development	Participants Involved	Purpose of the Step	Type of Validity Assessed
1	Forward translation	Two independent bilingual professional translators (Portuguese native speakers)	Initial translation of the instrument from English to Portuguese	—
2	Synthesis I	Two forward translators, 1 additional independent bilingual and bicultural professional medical translator, 2 research team members	Reconciliation of forward translations and synthesis into a preliminary initial translated version	—
3	Blind back-translation	Two independent bilingual professional translators (English native speakers)	Translation of the Portuguese version back into English to check for semantic equivalence	—
4	Synthesis II (multidisciplinary committee review)	One methodologist, 1 health care professional (both from the research team), all translators	Comparison of back-translations with the original version; conceptual, semantic, and content equivalence	Conceptual, semantic, and content equivalence
5	Pilot test (expert panel)	Ten experts (family doctors and researchers with experience in decision aids and validation), moderated by a member of the research team	Assessment of clarity and content relevance of the translated items using quantitative and qualitative methods	Content validity (clarity, content relevance, I-CVI, S-CVI/Ave, Fleiss’ kappa)

I-CVI, Item-Level Content Validity Index; IPDAS, International Patient Decision Aid Standards; S-CVI/Ave, Scale-Level Content Validity Index.

#### Step 1: Forward translation

In the first stage, 2 forward translations of the instruments from the original language (English) to the target language (the language to which we were translating the instrument, Portuguese, in this case) were produced by 2 independent bilingual professional translators whose mother tongue was Portuguese. The translators had different backgrounds: one was a specialized medical translator, knowledgeable about health care terminology and the content area of the instrument construct, and the other was neither knowledgeable about medical terminology nor familiar with the construct of the instrument. Both translators sought to reflect the usual spoken language and cultural nuances of the general population. From these translations, 2 versions were obtained: Translation 1 (TL1) and Translation 2 (TL2).

#### Step 2: Synthesis I

A third independent bilingual and bicultural professional medical translator compared TL1 with TL2 as well as both translations with the original version of the instrument. Using a committee approach, the 2 translators of step 1, the translator from this step (step 2), and 2 members of the research team met via Zoom to discuss any ambiguities and discrepancies and synthesize the results of the 2 translations. They then produced a preliminary initial translated version (PI-TL) of the instrument.

#### Step 3: Blind back-translation

Working from the PI-TL of the questionnaire and blinded to the original version, 2 independent professional translators (whose mother tongue was English, the language of the source instrument) translated the questionnaire back into their original language, producing 2 back-translated versions of the instrument (B-TL1 and B-TL2). The 2 translators had different backgrounds: one specialized in medical translation (and back-translation) while the other was not familiar with medical translation.

The 2 translators who performed the back-translations in step 3 were different from those who performed the initial forward translations in step 1. This ensured independent evaluation and minimized the risk of bias.

#### Step 4: Synthesis II

At this stage, a multidisciplinary committee compared the 2 back-translations of the instrument with each other and with the original instrument during a meeting held on Zoom. Following the recommendations of Sousa and Rojjanasrirat,^14^ the multidisciplinary committee consisted of 1 methodologist (a member of the research team), 1 health care professional familiar with the domains of the instrument’s construct (also a member of the research team), and all translators involved in steps 1 to 3. The committee’s role was to evaluate the conceptual, semantic, and content equivalence of the back-translated instruments and develop a prefinal version of the instrument (P-FTL) for pilot testing and psychometric testing.

#### Step 5: Pilot test of the prefinal version (cognitive debriefing)

This phase ensured that the adapted version retained its equivalence in the applied situation. To determine the conceptual equivalence of the translated instrument, an expert panel comprising 10 members knowledgeable about the content areas of the instrument construct and whose mother language was Portuguese evaluated the quantitative and qualitative content validity of the P-FTL.

A letter of invitation to participate in the expert panel was sent via e-mail to each potential member. This panel included 2 family doctors with more than 5 y of practice, 2 family doctors with less than 5 y of practice, 3 researchers who had published at least 1 paper related to PDAs, and 3 researchers with experience in translating, adapting, and validating instruments.

Two weeks before the meeting, the authors sent the English and prefinal Portuguese versions of the IPDASi, a document with scales to evaluate the clarity and content relevance of the instrument, and a Google Form questionnaire to record their responses to the members who accepted the invitation (Appendix 2).

The questionnaire used in the pilot test to evaluate content validity comprised 2 sections: 1) an introductory section explaining the objectives of this step, followed by a clarity quantitative assessment question for each criterion and an open-ended question for qualitative comments and 2) a section with the scale to assess the content relevance of the instrument, again followed by an open comment box.

To evaluate the clarity of the P-FTL items, each expert rated the instrument criteria as clear or unclear. Then, the percentage agreement and Fleiss’ kappa values were calculated. The minimum interrater agreement for the sample was defined as 80%. Criteria found to be “unclear” by at least 20% of the panel were flagged for revision. These items were discussed and adjusted accordingly during the expert panel meeting.^19^ To further evaluate the consistency of the expert ratings, Fleiss’ kappa was calculated. This statistic measures interrater agreement beyond what would be expected by chance and is particularly suitable when more than 2 raters are involved. The interpretation of kappa values typically follows the Landis and Koch classification: values less than 0.20 indicate slight agreement, 0.21 to 0.40 fair, 0.41 to 0.60 moderate, 0.61 to 0.80 substantial, and greater than 0.80 almost perfect agreement. In the content validity studies, kappa values greater than 0.60 are generally considered acceptable, although values greater than 0.75 are preferred for strong agreement.^20^

The expert panel was also asked to evaluate each item of the instrument for content relevance using a 4-point Likert-type scale: 1 = *not relevant*, 2 = *somewhat relevant—item needs some revision*, 3 = *quite relevant but needs minor revision*, and 4 = *very relevant*. The expert panel was instructed to compare the Portuguese version of each item with the original English version, focusing on both semantic equivalence and cultural appropriateness. Ratings of 1 or 2 were considered content invalid, while ratings of 3 or 4 were considered content valid. Items classified as 1 or 2 by any panel member were considered indications that an item required revision. All such items were discussed and revised during the expert panel meeting.^21,22^

To quantify content validity, the Content Validity Mean Score, the Item-Level Content Validity Index (I-CVI), and Scale-Level Content Validity Index (S-CVI/Ave) were calculated. The Content Validity Mean Score was calculated as the mean score for each item across all reviewers. This mean score is not a psychometric indicator in itself but provides an additional descriptive measure of how each item was perceived by the panel. The Content Validity Indices reflect the proportion of experts who consider each item relevant. The I-CVI was calculated for each criterion as the number of experts giving a rating of 3 or 4 for each item (i.e., content valid) divided by the total number of experts of the expert panel. Values range from 0 to 1, where I-CVI >0.79 indicates the item is relevant and between 0.70 and 0.79 means the item needs revisions; and if the value is <0.70, the item should be eliminated.^22^ S-CVI/Ave was then computed as the average of all I-CVI values. A value ≥0.90 was considered indicative of excellent overall content validity. An I-CVI ≥0.78 and S-CVI/Ave ≥0.90 were the minimum acceptable indices.^21–23^

To increase confidence in the content validity of the instrument, Fleiss’ kappa was also determined for relevance ratings.

Finally, a qualitative assessment was conducted during the expert panel meeting. Members were asked to compare each translated item to the original in terms of meaning, clarity, and cultural relevance. They were encouraged to reflect on grammar, vocabulary, and overall phrasing and to suggest improvements where needed. Discrepancies in interpretation or suggested changes were resolved through discussion and consensus building among the panel members, moderated by a member of the research team. This process ensured that both the linguistic and conceptual integrity of the instrument were maintained in the Portuguese context.

### Sample Size and Sampling

The research team used an expert panel composed of 10 members for the pilot test of the prefinal version of the translated IPDASi (step 5). The literature recommends using an expert panel composed of 6 to 10 members to evaluate the clarity of the items and content equivalence of the translated instrument.^21,23^

### Data Collection Methods and Sources

The translation and cultural adaptation process occurred from September to December 2023. Data collection from the pilot test occurred from January to February 2024 and was then entered into a computer database.

The Synthesis meetings involved the translators and 2 members of the research team and were used to resolve discrepancies and ensure cultural adaptation of the instrument. The expert panel, composed of 10 members not involved in the earlier steps, was responsible for evaluating the clarity and content relevance of the translated instrument. Quantitative data were recorded using a structured online Google Form, while qualitative feedback was captured through open-ended comments and interactive discussions during the final expert panel meeting.

Synthesis I, Synthesis II, and pilot test meetings were held via the Zoom platform. Participants gave verbal consent to participate at the beginning of each Zoom meeting.

### Data Analysis

Quantitative variables were described by the mean, standard deviation, and minimum and maximum values ( $\bar{x}$ ±*s*, min, max), if normally distributed, or by the median and respective interquartile interval [Q_1_; Q_3_], if nonnormally distributed. The normality of the variables was analyzed by observing the respective histograms. Data analyses were performed using SPSS Statistics version 27^®^.

To evaluate the clarity of each item, a dichotomous scale (clear or unclear) was used. The overall and specific percentage agreements and Fleiss’ kappa were then calculated with the corresponding 95% confidence interval (CI). To evaluate the content validity of the instrument, we used both the I-CVI and the S-CVI/Ave, along with Fleiss’ kappa statistic, with the respective 95% CI.

Qualitative data analysis occurred during the expert panel meeting. All 10 panel members were asked to examine each criterion in the Portuguese version alongside its original English version. They provided feedback on semantic equivalence, cultural appropriateness, grammar, and phrasing. When discrepancies or uncertainties arose, they were discussed openly among the panel until consensus was reached. This structured discussion ensured alignment with best practices for cognitive debriefing and qualitative validation in instrument adaptation.

### Patient and Public Involvement

Patients and the public were not involved, given that the IPDAS is primarily used by researchers and health care professionals.

### Role of the Funding Source for the Study

The funding source had no role in the study design; data collection, analysis, or interpretation; manuscript preparation; or the decision to submit the manuscript for publication. The authors declare that this research was conducted independently of any influence from funding organizations.

## Results

### Translation and Cultural Adaptation of the Instrument (Steps 1 to 4)

During the Synthesis I Zoom meeting, the PI-TL was analyzed, and several ambiguities were addressed. For instance, feedback received during the meeting highlighted that some terms were direct translations from English and required adjustments. An example of this is the term “qualifications,” which was translated literally as *qualificações*; however, the correct term used by Portuguese speakers is *habilitações*. This change improved the wording of some items, enhancing their clarity and linguistic adequacy. Appendix 3 contains all the changes made at the Synthesis I meeting.

At the Synthesis II meeting, when the multidisciplinary committee compared the 2 back-translations of the instrument with each other and with the original instrument, no discrepancies were observed. As a result, no further changes were made to the PI-TL, and the P-FTL of the instrument was then ready for pilot testing.

### Validation of the Instrument (Step 5: Pilot Test of the Prefinal Version)

At the expert panel meeting, each of the 44 criteria were individually analyzed, discussed, and commented on. The panel sought to adapt each criterion to the linguistic specificities of Portuguese from Portugal and successfully reached a consensus solution for all items of the instrument under analysis. The quantitative measures of validity are summarized in Tables 2 and 3.

**Table 2:** Expert Panel Quantitative Measures of Clarity

	Criterion	Clear %	Fleiss’ Kappa (95% CI)	Overall Agreement	Specific Agreement
Qualifying criteria	Q1	90	0.033 [−0.08; 0.14]	0.92 [0.87; NA]	Clear: 0.96 [0.93; 1] Unclear: 0.07 [0; 0.11]
	Q2	100			
	Q3	100			
	Q4	100			
	Q5	100			
	Q6	100			
	Q7	80			
Certification criteria	C1	100	0.045 [−0.05; 0.14]	0.94 [0.89; NA]	Clear: 0.97 [0.94; 1] Unclear: 0.07 [0; 0.11]
	C2	90			
	C3	100			
	C4	100			
	C5	100			
	C6	100			
	C7	100			
	C8	100			
	C9	100			
	C10	80			
Quality criteria	QU1	90	0.031 [−0.03; 0.09]	0.93 [0.89; 0.97]	Clear: 0.96 [0.94; 0.99] Unclear: 0.07 [0; 0.10]
	QU2	100			
	QU3	100			
	QU4	80			
	QU5	100			
	QU6	100			
	QU7	90			
	QU8	100			
	QU9	100			
	QU10	100			
	QU11	100			
	QU12	100			
	QU13	80			
	QU14	80			
	QU15	100			
	QU16	100			
	QU17	100			
	QU18	100			
	QU19	90			
	QU20	100			
	QU21	90			
	QU22	100			
	QU23	100			
	QU24	100			
	QU25	100			
	QU26	100			
	QU27	100			
	Total		0.03 [−0.01; 0.08]	0.93 [0.89; 0.97]	Clear: 0.96 [0.94; 0.98] Unclear: 0.07 [0.03; 0.10]

CI, confidence interval.

**Table 3:** Expert Panel Quantitative Measures of Content Validity

	Criterion	Content Validity Mean Score	I-CVI	Fleiss’ Kappa [95% CI]
Qualifying Criteria	Q1	3.90 (0.32)	1.00	−0.03 [−0.14; 0.08]
	Q2	4 (0)	1.00	
	Q3	4 (0)	1.00	
	Q4	4 (0)	1.00	
	Q5	4 (0)	1.00	
	Q6	4 (0)	1.00	
	Q7	3.90 (0.32)	1.00	
Certification Criteria	C1	3.90 (0.32)	1.00	−0.03 [−0.11; 0.05]
	C2	3.70 (0.48)	1.00	
	C3	3.90 (0.32)	1.00	
	C4	3.70 (0.68)	0.90	
	C5	4 (0)	1.00	
	C6	3.8 (0.42)	1.00	
	C7	4 (0)	1.00	
	C8	3.90 (0.32)	1.00	
	C9	3.90 (0.32)	1.00	
	C10	3.70 (0.68)	0.90	
Quality Criteria	QU1	3.90 (0.32)	1.00	−0.04 [−0.08; 0.01]
	QU2	4 (0)	1.00	
	QU3	3.90 (0.32)	1.00	
	QU4	3.60 (0.97)	0.90	
	QU5	3.90 (0.32)	1.00	
	QU6	3.90 (0.32)	1.00	
	QU7	3.80 (0.42)	1.00	
	QU8	3.90 (0.32)	1.00	
	QU9	3.90 (0.32)	1.00	
	QU10	3.40 (0.97)	0.90	
	QU11	3.30 (1.25)	0.80	
	QU12	3.90 (0.32)	1.00	
	QU13	3.90 (0.32)	1.00	
	QU14	3.90 (0.32)	1.00	
	QU15	4 (0)	1.00	
	QU16	4 (0)	1.00	
	QU17	3.80 (0.42)	1.00	
	QU18	3.80 (0.42)	1.00	
	QU19	3.80 (0.63)	0.90	
	QU20	3.30 (1.06)	0.80	
	QU21	3.90 (0.32)	1.00	
	QU22	3.80 (0.42)	1.00	
	QU23	3.60 (0.97)	0.90	
	QU24	3.60 (0.97)	0.90	
	QU25	3.60 (0.97)	0.90	
	QU26	3.60 (0.97)	0.90	
	QU27	3.90 (0.32)	1.00	
	Total			−0.02 [−0.06; 0.02]
			S-CVI/Ave 0.97	

CI, confidence interval; I-CVI, Item-Level Content Validity Index.

Criteria Q7, C10, QU4, QU13, and QU14 were found to be unclear by at least 20% of the panel and were revised and reevaluated during the expert panel meeting. Changes to these criteria are listed in Table 4. The overall agreement was 0.93 [0.89; 0.97], and the calculated Fleiss’ kappa was 0.03 [−0.01; 0.08].

**Table 4:** Unclear Criteria Changes during the Pilot Test Step

Criterion	Changes made
**Q7:** The patient decision aid describes what it is like to experience the consequences of the options (e.g., physical, psychological, social) and/or provides an explicit values clarification exercise that asks patients to consider or rate which positive and negative features of the options matter most to them.	There was some concern about the length of this criterion. After group discussion, and since it was a suggestion from the IPDAS Steering Committee to add the option of an explicit values clarification exercise to this criterion, we decided to keep it as it was. We also decided to change the word “client” to “*paciente*” throughout the document, since it is a more correct translation of “patient.”
**C10:** The patient decision aid has information about the consequences of detecting the condition or disease that would never have caused problems if screening had not been done (lead time bias).	It was decided to change “lead time bias” to “overdiagnosis bias.”
**QU4:** The patient decision aid specifies the event rates for the outcome probabilities.	There was much discussion regarding this criterion. The panel members found the meaning of the phrase unclear, even in the original instrument. Additional clarification on this criterion was requested from the original authors. Following this clarification, the research team decided to add the following explanation to the criterion: “(for example, states that ‘X out of 100 people will develop’ the outcome, instead of vague terms like ‘some people will develop’).” It was also decided to retain the word “outcome” in English, as it is the most used term in Portugal.
**QU13:** The development process included review by clients/patients not involved in producing the decision support intervention.	It was decided to change “decision support intervention” to “patient decision aid” to make the criterion clearer. In addition, the term “clients” was removed, since it is not commonly used in Portugal.
**QU14:** The development process included review by professionals not involved in producing the decision support intervention.	It was decided to change “decision support intervention” to “patient decision aid” to make the criterion clearer.

Eleven criteria (C4, C10, QU4, QU10, QU11, QU19, QU20, QU23, QU24, QU25, and QU26) were rated as content invalid by at least 1 panel member (i.e., received a score of 1 or 2 on the 4-point relevance scale) and were revised during the expert panel meeting. In this context, “content invalid” indicates that the item was perceived as lacking sufficient relevance or requiring substantial modification to improve semantic equivalence and cultural appropriateness in the Portuguese version.

The Content Validity Mean Score ranged from 3.30 ± 1.06 to 4.00 ± 0.00. The I-CVI score was >0.79 for all criteria. The S-CVI calculated for the entire instrument was 0.97, and the calculated Fleiss’ kappa was −0.02 [−0.06; 0.02].

The final Portuguese version of the IPDASi is shown in Table 5.

**Table 5:** Portuguese Version of the IPDAS Minimal Criteria v4.0 (2013) Instrument

Versão portuguesa do instrumento IPDASi (versão 4.0) International Patient Decision Aid Standards instrument (IPDASi) v.4.0 (2013)			
**Critérios de qualificação:** critérios considerados de carácter de definição. Para serem consideradas para certificação e classificadas como auxiliar de decisão para o paciente, as ferramentas deverão satisfazer todos os critérios de qualificação.			
	**Critério**	**Cumpre o critério**	
Q1	O auxiliar de decisão descreve a doença ou o procedimento de saúde (tratamento, procedimento ou exame) para o qual é necessária a decisão em causa.	Sim	Não
Q2	O auxiliar de decisão indica explicitamente a decisão que terá de ser considerada (decisão em causa).	Sim	Não
Q3	O auxiliar de decisão identifica o público-alvo.	Sim	Não
Q4	O auxiliar de decisão descreve as opções disponíveis para a decisão em causa.	Sim	Não
Q5	O auxiliar de decisão descreve os aspetos positivos (benefícios ou vantagens) de cada opção.	Sim	Não
Q6	O auxiliar de decisão descreve as características negativas (danos, efeitos indesejáveis ou desvantagens) de cada opção.	Sim	Não
Q7	O auxiliar de decisão descreve o impacto das consequências das opções (por exemplo, físicas, psicológicas, sociais) e/ou inclui um exercício de valorização explícito que pede aos pacientes para ponderarem ou classificarem os aspetos positivos ou negativos que são mais importantes para eles.	Sim	Não
**Critérios de certificação:** critérios que são essenciais para evitar o risco de uma decisão enviesada. As ferramentas devem cumprir todos os critérios de certificação para cumprir as normas de certificação.			
	**Critério**	**Cumpre o critério**	
C1	O auxiliar de decisão apresenta de forma semelhante os aspetos positivos e negativos das opções (por exemplo, usando tipos de letra iguais, mesma sequência, mesma apresentação de informações estatísticas).	Sim	Não
C2	O auxiliar de decisão (ou a documentação associada) inclui as citações da evidência utilizada.	Sim	Não
C3	O auxiliar de decisão (ou a documentação associada) inclui uma data de redação ou publicação.	Sim	Não
C4	O auxiliar de decisão (ou a documentação associada) inclui informações sobre a política de atualização.	Sim	Não
C5	O auxiliar de decisão inclui informações sobre os níveis de incerteza quanto à probabilidade de um determinado evento ou outcome (por exemplo, indicando um intervalo ou utilizando afirmações como “na nossa melhor estimativa […]”).	Sim	Não
C6	O auxiliar de decisão (ou a documentação associada) inclui informações sobre o financiamento usado para desenvolver o auxiliar de decisão.	Sim	Não
C7	O auxiliar de decisão descreve aquilo que o teste se destina a avaliar.	Sim	Não
C8	Se o teste detetar a doença ou o problema de saúde, o auxiliar de decisão descreve os próximos passos habituais.	Sim	Não
C9	O auxiliar de decisão descreve os próximos passos, se a doença ou o problema de saúde não for detetado.	Sim	Não
C10	O auxiliar de decisão inclui informações sobre as consequências de detetar um problema de saúde ou doença que nunca teria causado problemas caso não tivesse sido feito um rastreio (sobrediagnóstico).	Sim	Não
**Critérios de qualidade:** critérios que são desejáveis porque vão melhorar um auxiliar de decisão, mas que não são essenciais para reduzir o risco de uma decisão enviesada. Estes aspetos melhorariam a experiência de utilização do auxiliar de decisão, mas não seria expectável que a inexistência dos mesmos influenciasse a decisão de uma pessoa de forma negativa.			
	**Critério**	**Cumpre o critério**	
QU1	O auxiliar de decisão descreve a história natural da doença ou problema de saúde se não for tomada qualquer medida (quando apropriado).	Sim	Não
QU2	O auxiliar de decisão possibilita a comparação entre os aspetos positivos e negativos das opções disponíveis.	Sim	Não
QU3	O auxiliar de decisão inclui informações sobre as probabilidades dos outcomes associadas às opções (ou seja, as consequências prováveis das decisões).	Sim	Não
QU4	Dadas as probabilidades dos outcomes, o auxiliar de decisão especifica as taxas de ocorrência dos mesmos (por exemplo, refere que “X em cada 100 pessoas irá desenvolver” o outcome, em vez de termos vagos como “algumas pessoas irão desenvolver”).	Sim	Não
QU5	O auxiliar de decisão permite ao utilizador comparar as probabilidades dos outcomes de várias opções no mesmo período de tempo (quando possível).	Sim	Não
QU6	O auxiliar de decisão permite ao utilizador comparar as probabilidades dos outcomes de várias opções usando o mesmo denominador (quando possível).	Sim	Não
QU7	O auxiliar de decisão possibilita visualizar as probabilidades dos outcomes de mais do que uma forma (por exemplo, em texto, números e diagramas).	Sim	Não
QU8	O auxiliar de decisão pede aos pacientes que pensem sobre quais os aspetos positivos e negativos das opções são mais importantes para eles (de forma implícita ou explícita).	Sim	Não
QU9	O auxiliar de decisão inclui um método passo-a-passo para a tomada de decisão.	Sim	Não
QU10	O auxiliar de decisão inclui ferramentas como fichas de trabalho ou listas de perguntas para utilizar ao discutir as opções com um profissional de saúde.	Sim	Não
QU11	O processo de desenvolvimento incluiu uma avaliação de necessidades com os pacientes.	Sim	Não
QU12	O processo de desenvolvimento incluiu uma avaliação de necessidades com os profissionais de saúde.	Sim	Não
QU13	O processo de desenvolvimento incluiu a revisão por pacientes não envolvidos na produção do auxiliar de decisão.	Sim	Não
QU14	O processo de desenvolvimento incluiu a revisão por profissionais não envolvidos na produção do auxiliar de decisão.	Sim	Não
QU15	O auxiliar de decisão foi testado na prática com pacientes que tinham de tomar a decisão.	Sim	Não
QU16	O auxiliar de decisão foi testado na prática com profissionais de saúde que aconselham pacientes que estão perante a decisão.	Sim	Não
QU17	O auxiliar de decisão (ou a documentação associada) descreve a forma como a evidência científica foi selecionada ou resumida.	Sim	Não
QU18	O auxiliar de decisão (ou a documentação associada) descreve a qualidade da evidência científica utilizada.	Sim	Não
QU19	O auxiliar de decisão inclui as habilitações dos autores/responsáveis pelo desenvolvimento da ferramenta.	Sim	Não
QU20	O auxiliar de decisão (ou a documentação associada) indica os níveis de legibilidade (utilizando 1 ou mais das escalas disponíveis).	Sim	Não
QU21	Existe evidência de que o auxiliar de decisão aumenta a correspondência entre as preferências do doente informado e a opção selecionada.	Sim	Não
QU22	Existe evidência de que o auxiliar de decisão ajuda a aumentar o conhecimento dos pacientes sobre as características das opções existentes.	Sim	Não
QU23	O auxiliar de decisão inclui informações sobre a probabilidade de um teste ter um resultado verdadeiro positivo.	Sim	Não
QU24	O auxiliar de decisão inclui informações sobre a probabilidade de um teste ter um resultado verdadeiro negativo.	Sim	Não
QU25	O auxiliar de decisão inclui informações sobre a probabilidade de um teste ter um resultado falso positivo.	Sim	Não
QU26	O auxiliar de decisão inclui informações sobre a probabilidade de um teste ter um resultado falso negativo.	Sim	Não
QU27	O auxiliar de decisão descreve a probabilidade de a doença ser detetada com e sem a utilização do teste.	Sim	Não

IPDAS, International Patient Decision Aid Standards.

Original: IPDAS Minimal Criteria v4.0 (2013) instrument. Traduzido com autorização dos autores originais.

Autores originais: Natalie Joseph-Williams, Robert Newcombe, Mary Politi, Marie-Anne Durand, Stephanie Sivell, Dawn Stacey, Annette O’Connor, Robert J. Volk, Adrian Edwards, Carol Bennett, Michael Pignone, Richard Thomson, Glyn Elwyn.

Referência: Toward minimum standards for certifying patient decision aids: a modified Delphi consensus process. *Med Decis Making*. 2014;34(6):699–710. DOI: 10.1177/0272989X13501721

Traduzido por: Micaela Gregório, Andreia Teixeira, Mariana Teixeira, Inês Marques, Ana Sofia Correia, Phillippa Bennett, Helen Carter, Dawn Stacey, Carlos Martins.

Data de aprovação como versão portuguesa: 05/03/2024

## Discussion

This study contributes new evidence regarding the translation, cultural adaptation, and content validation of the IPDAS Minimal Criteria v4.0 (2013) for use in the Portuguese context. The translation process followed a structured methodology, including forward and backward translation, multidisciplinary committee review, and pilot testing with an expert panel. Although our findings support the semantic and cultural relevance of the Portuguese version, we acknowledge that this study focused on linguistic and contextual adaptation rather than broader frameworks of cultural adaptation.

The cultural adaptation process played a crucial role in enhancing item clarity and contextual appropriateness. For instance, the term “*qualificações*” (a literal translation of “qualifications”) was replaced with “*habilitações*,” which is more commonly used in Portugal to refer to educational background. Similarly, technical or ambiguous phrases were reworded to reflect plain language more accessible to end users, ensuring that the adapted instrument better reflects local idioms and cultural norms. This adaptation underscores the importance of engaging native speakers and experts in the translation process to preserve both conceptual and practical relevance.

Although the expert panel reached high overall agreement on item clarity and relevance, Fleiss’ kappa values were low. This apparent contradiction is not unusual in validation studies using dichotomous or ordinal rating scales. Fleiss’ kappa measures interrater agreement beyond chance and is known to be sensitive to the prevalence of ratings within categories. In our case, the predominance of ratings such as “clear” or “very relevant”—indicating high agreement—reduced kappa values due to limited variability. According to common interpretative thresholds,^20^ our values would be considered poor (<0.20); however, when combined with high percentage agreement and content validity indices, they offer a more nuanced and reassuring picture of item quality.^24,25^

The expert panel identified several unclear criteria that were revised and reevaluated, underscoring the importance of expert input. The revised items did not change the meaning of the original items.

Eleven criteria were initially rated as content invalid by at least 1 expert, meaning they were scored as either “not relevant” or “somewhat relevant—needs revision.” These items were carefully discussed and revised during the expert panel meeting. Following revisions, all criteria achieved acceptable content validity scores, as reflected in high I-CVI and S-CVI/Ave indices. This supports the appropriateness of iterative refinement based on expert feedback. Nonetheless, the limited variability in response categories on the relevance scale likely contributed again to the low Fleiss’ kappa for content validity.

Although this study focused on the linguistic and cultural adaptation of the instrument for European Portuguese, the translated tool may serve as a foundation for future adaptations in other Portuguese-speaking regions. Further validation would be necessary to ensure appropriateness for Brazilian Portuguese speakers, given important lexical and contextual differences.

## Conclusion

This study provides initial evidence supporting the semantic and contextual relevance of a Portuguese version of the IPDAS Minimal Criteria instrument. Through a structured process of translation, adaptation, and expert review, the instrument demonstrated strong content validity and high agreement regarding clarity and relevance of its items. While the results are encouraging, the scope of this study was limited to linguistic and contextual adaptation; broader aspects of cultural adaptation were not evaluated.

The findings of this study demonstrate the critical role of expert panels in the translation and cultural adaptation of instruments such as the IPDASi. Despite limitations in interrater agreement due to methodological constraints of the rating scales used, the combined results from content validity indices and agreement measures support the use of this instrument for evaluating PDAs in Portugal.

This study addresses the significant gap in the availability of high-quality decision aids in European Portuguese to facilitate better patient involvement in health care decisions. Future studies should focus on broader validation efforts to establish the psychometric properties of the Portuguese IPDASi and ensure its reliability and validity in practice.

Not applicable.

## Supplemental Material

sj-docx-1-mpp-10.1177_23814683251386451 – Supplemental material for Translation, Cultural Adaptation, and Validation of the International Patient Decision Aid Standards Minimal Criteria Instrument for the Portuguese Population Supplemental material, sj-docx-1-mpp-10.1177_23814683251386451 for Translation, Cultural Adaptation, and Validation of the International Patient Decision Aid Standards Minimal Criteria Instrument for the Portuguese Population by Micaela Gregório, Andreia Teixeira, Mariana Teixeira, Inês Marques, Ana Sofia Correia, Phillippa May Bennett, Helen Carter, Dawn Stacey and Carlos Martins in MDM Policy & Practice
