# Supplementary material for: Translation, Cultural Adaptation, and Validation of the International Patient Decision Aid Standards Minimal Criteria Instrument for the Portuguese Population
Source: MDM Policy Pract. 2025 Oct 18;10(2):23814683251386451. doi: 10.1177/23814683251386451 (PMC12541180; doi:10.1177/23814683251386451)
Supplement: sj-docx-1-mpp-10.1177_23814683251386451 – Supplemental material for Translation, Cultural Adaptation, and Validation of the International Patient Decision Aid Standards Minimal Criteria Instrument for the Portuguese Population [file sj-docx-1-mpp-10.1177_23814683251386451.docx]

**SUPPLEMENTARY MATERIAL**

**APPENDIX 1 – English version of the “IPDAS Minimal criteria v4.0 (2013) instrument”**

**International Patient Decision Aid Standards instrument (IPDASi) - Version 4.0**

- **Qualifying Criteria:** criteria that are considered definitional in nature. To be considered for certification and be classified as a patient decision aid, tools should meet all qualifying criteria.

|  | Criteria | Meets criteria | |
| --- | --- | --- | --- |
| Q1 | The patient decision aid describes the health condition or problem (treatment, procedure, or investigation) for which the index decision is required. | Yes | No |
| Q2 | The patient decision aid explicitly states the decision that needs to be considered (index decision). | Yes | No |
| Q3 | The patient decision aid identifies the target audience. | Yes | No |
| Q4 | The patient decision aid describes the options available for the index decision. | Yes | No |
| Q5 | The patient decision aid describes the positive features (benefits or advantages) of each option. | Yes | No |
| Q6 | The patient decision aid describes the negative features (harms, side effects, or disadvantages) of each option. | Yes | No |
| Q7 | The patient decision aid describes what it is like to experience the consequences of the options (e.g., physical, psychological, social) and/or provides an explicit values clarification exercise that asks patients to consider or rate which positive and negative features of the options matter most to them. | Yes | No |

- **Certification Criteria:** criteria that are essential in order to avoid risk of harmful bias. Tools should meet all certification criteria to reach certification standards.

|  | Criteria | Meets criteria | |
| --- | --- | --- | --- |
| C1 | The patient decision aid shows the negative and positive features of options with equal detail (e.g., using similar fonts, sequence, presentation of statistical information). | Yes | No |
| C2 | The patient decision aid (or associated documentation) provides citations to the evidence selected. | Yes | No |
| C3 | The patient decision aid (or associated documentation) provides a production or publication date. | Yes | No |
| C4 | The patient decision aid (or associated documentation) provides information about the update policy. | Yes | No |
| C5 | The patient decision aid provides information about the levels of uncertainty around event or outcome probabilities (e.g., by giving a range or by using phases such as ‘‘our best estimate is...’’). | Yes | No |
| C6 | The patient decision aid (or associated documentation) provides information about the funding source used for development. | Yes | No |
| C7 | The patient decision aid describes what the test is designed to measure. | Yes | No |
| C8 | If the test detects the condition or problem, the patient decision aid describes the next steps typically taken. | Yes | No |
| C9 | The patient decision aid describes the next steps if the condition or problem is not detected. | Yes | No |
| C10 | The patient decision aid has information about the consequences of detecting the condition or disease that would never have caused problems if screening had not been done (lead time bias). | Yes | No |

- **Quality Criteria:** criteria that are desirable because they will enhance a decision aid but are not essential for reducing risk of harmful bias. These items would improve the experience of using the decision aid, but absence of the item would not be expected to influence the individual’s decision in a negative way.

|  | Criteria | Meets criteria | |
| --- | --- | --- | --- |
| QU1 | The patient decision aid describes the natural course of the health condition or problem, if no action is taken (when appropriate). | Yes | No |
| QU2 | The patient decision aid makes it possible to compare the positive and negative features of the available options. | Yes | No |
| QU3 | The patient decision aid provides information about outcome probabilities associated with the options (i.e., the likely consequences of decisions). | Yes | No |
| QU4 | The patient decision aid specifies the event rates for the outcome probabilities. | Yes | No |
| QU5 | The patient decision aid allows the user to compare outcome probabilities across options using the same time period (when feasible). | Yes | No |
| QU6 | The patient decision aid allows the user to compare outcome probabilities across options using the same denominator (when feasible). | Yes | No |
| QU7 | The patient decision aid provides more than 1 way of viewing the probabilities (e.g., words, numbers, and diagrams). | Yes | No |
| QU8 | The patient decision aid asks patients to think about which positive and negative features of the options matter most to them (implicitly or explicitly). | Yes | No |
| QU9 | The patient decision aid provides a step-by-step way to make a decision. | Yes | No |
| QU10 | The patient decision aid includes tools like worksheets or lists of questions to use when discussing options with a practitioner. | Yes | No |
| QU11 | The development process included a needs assessment with clients or patients. | Yes | No |
| QU12 | The development process included a needs assessment with health professionals. | Yes | No |
| QU13 | The development process included review by clients/patients not involved in producing the decision support intervention. | Yes | No |
| QU14 | The development process included review by professionals not involved in producing the decision support intervention. | Yes | No |
| QU15 | The patient decision aid was field tested with patients who were facing the decision. | Yes | No |
| QU16 | The patient decision aid was field tested with practitioners who counsel patients who face the decision. | Yes | No |
| QU17 | The patient decision aid (or associated documentation) describes how research evidence was selected or synthesised. | Yes | No |
| QU18 | The patient decision aid (or associated documentation) describes the quality of the research evidence used. | Yes | No |
| QU19 | The patient decision aid includes authors’/ developers’ credentials or qualifications. | Yes | No |
| QU20 | The patient decision aid (or associated documentation) reports readability levels (using 1 or more of the available scales). | Yes | No |
| QU21 | There is evidence that the patient decision aid improves the match between the preferences of the informed patient and the option that is chosen. | Yes | No |
| QU22 | There is evidence that the patient decision aid helps patients improve their knowledge about options’ features. | Yes | No |
| QU23 | The patient decision aid includes information about the chances of having a true-positive test result. | Yes | No |
| QU24 | The patient decision aid includes information about the chances of having a true-negative test result. | Yes | No |
| QU25 | The patient decision aid includes information about the chances of having a false-positive test result. | Yes | No |
| QU26 | The patient decision aid includes information about the chances of having a false-negative test result. | Yes | No |
| QU27 | The patient decision aid describes the chances the disease is detected with and without the use of the test. | Yes | No |

**Authors:** Natalie Joseph-Williams, Robert Newcombe, Mary Politi, Marie-Anne Durand, Stephanie Sivell, Dawn Stacey, Annette O’Connor, Robert J. Volk, Adrian Edwards, Carol Bennett, Michael Pignone, Richard Thomson, Glyn Elwyn.

**Reference:** Toward Minimum Standards for Certifying Patient Decision Aids: A Modified Delphi Consensus Process. Med Decis Mak Int J Soc Med Decis Mak. 2014 Aug;34(6):699–710. DOI: 10.1177/0272989X13501721

**APPENDIX 2 – Document sent to the Expert Panel members in Step 5.**

**Translation, Cultural Adaptation and Validation of the “IPDAS Minimal Criteria v.4.0 (2013) instrument” for the Portuguese language**

**Step 5: Pilot-test of the pre-final version (cognitive debriefing)**

Nesta fase do processo, pretende-se realizar o pré-teste da versão traduzida do “IPDAS Minimal Criteria v.4.0 (2013) instrument” (IPDASi).

Para isso, de forma a podermos avaliar a clareza e a validade de conteúdo da versão portuguesa do instrumento, pedimos-lhe que preencha o seguinte documento, classificando cada um dos critérios segundo as escalas abaixo.

Relativamente à clareza dos critérios, pedimos que sempre que classifique um critério como “Não claro”, deixe na secção dos “Comentários” uma sugestão sobre como reescrever o critério de forma a tornar a linguagem mais clara.

Pode igualmente incluir comentários positivos e/ou sugestões de melhoria em cada um dos critérios, na respetiva secção de “Comentários”.

| Critério | | Clareza | Validade de conteúdo  1 = não relevante;  2 = pouco relevante – o critério necessita de alguma revisão;  3 = bastante relevante, mas necessita uma revisão minor;  4 = muito relevante. |
| --- | --- | --- | --- |
| Q1 | O auxiliar de decisão descreve a doença ou o problema de saúde (tratamento, procedimento ou exame) para o qual é necessária a decisão em causa. | Claro Não Claro  Comentários: | 1 2 3 4  Comentários: |
| Q2 | O auxiliar de decisão indica explicitamente a decisão que terá de ser considerada (decisão em causa). | Claro Não Claro  Comentários: | 1 2 3 4  Comentários: |
| Q3 | O auxiliar de decisão identifica o público-alvo. | Claro Não Claro  Comentários: | 1 2 3 4  Comentários: |
| Q4 | O auxiliar de decisão descreve as opções disponíveis para a decisão em causa. | Claro Não Claro  Comentários: | 1 2 3 4  Comentários: |
| Q5 | O auxiliar de decisão descreve os aspetos positivos (benefícios ou vantagens) de cada opção. | Claro Não Claro  Comentários: | 1 2 3 4  Comentários: |
| Q6 | O auxiliar de decisão descreve as características negativas (danos, efeitos indesejáveis ou desvantagens) de cada opção. | Claro Não Claro  Comentários: | 1 2 3 4  Comentários: |
| Q7 | O auxiliar de decisão descreve o impacto das consequências das opções (por exemplo, físicas, psicológicas, sociais) e/ou inclui um exercício de valorização explícito que pede aos doentes para ponderarem ou classificarem os aspetos positivos ou negativos que são mais importantes para eles. | Claro Não Claro  Comentários: | 1 2 3 4  Comentários: |
| C1 | O auxiliar de decisão apresenta os aspetos positivos e negativos das opções de forma semelhante (por exemplo, usando tipos de letra iguais, mesma sequência, mesma apresentação de informações estatísticas). | Claro Não Claro  Comentários: | 1 2 3 4  Comentários: |
| C2 | O auxiliar de decisão (ou a documentação associada) inclui citações da evidência selecionada. | Claro Não Claro  Comentários: | 1 2 3 4  Comentários: |
| C3 | O auxiliar de decisão (ou a documentação associada) inclui uma data de redação ou publicação. | Claro Não Claro  Comentários: | 1 2 3 4  Comentários: |
| C4 | O auxiliar de decisão (ou a documentação associada) inclui informações sobre a política de atualização. | Claro Não Claro  Comentários: | 1 2 3 4  Comentários: |
| C5 | O auxiliar de decisão inclui informações sobre os níveis de incerteza quanto à probabilidade de um determinado evento ou desfecho (por exemplo, indicando um intervalo ou utilizando afirmações como “na nossa melhor estimativa [...]”). | Claro Não Claro  Comentários: | 1 2 3 4  Comentários: |
| C6 | O auxiliar de decisão (ou a documentação associada) inclui informações sobre o financiamento usado para desenvolver a ferramenta. | Claro Não Claro  Comentários: | 1 2 3 4  Comentários: |
| C7 | O auxiliar de decisão descreve aquilo que o teste se destina a avaliar. | Claro Não Claro  Comentários: | 1 2 3 4  Comentários: |
| C8 | Se o teste detetar a doença ou o problema de saúde, o auxiliar de decisão descreve os próximos passos habituais. | Claro Não Claro  Comentários: | 1 2 3 4  Comentários: |
| C9 | O auxiliar de decisão descreve os próximos passos, se a doença ou o problema de saúde não for detetado. | Claro Não Claro  Comentários: | 1 2 3 4  Comentários: |
| C10 | O auxiliar de decisão inclui informações sobre as consequências de detetar um problema de saúde ou doença que nunca teria causado problemas caso não tivesse sido feito um rastreio (viés de antecipação diagnóstica). | Claro Não Claro  Comentários: | 1 2 3 4  Comentários: |
| QU1 | O auxiliar de decisão descreve o curso natural da doença ou problema de saúde se não for tomada qualquer medida (quando apropriado). | Claro Não Claro  Comentários: | 1 2 3 4  Comentários: |
| QU2 | O auxiliar de decisão possibilita a comparação entre os aspetos positivos e negativos das opções disponíveis. | Claro Não Claro  Comentários: | 1 2 3 4  Comentários: |
| QU3 | O auxiliar de decisão inclui informações sobre as probabilidades de desfecho associadas às opções (ou seja, as consequências prováveis das decisões). | Claro Não Claro  Comentários: | 1 2 3 4  Comentários: |
| QU4 | O auxiliar de decisão especifica as taxas de ocorrência do evento para as probabilidades do desfecho. | Claro Não Claro  Comentários: | 1 2 3 4  Comentários: |
| QU5 | O auxiliar de decisão permite ao utilizador comparar as probabilidades de desfecho de várias opções no mesmo período de tempo (quando possível). | Claro Não Claro  Comentários: | 1 2 3 4  Comentários: |
| QU6 | O auxiliar de decisão permite ao utilizador comparar as probabilidades de desfecho de várias opções usando o mesmo denominador (quando possível). | Claro Não Claro  Comentários: | 1 2 3 4  Comentários: |
| QU7 | O auxiliar de decisão possibilita visualizar as probabilidades de mais do que uma forma (por exemplo, em texto, números e diagramas). | Claro Não Claro  Comentários: | 1 2 3 4  Comentários: |
| QU8 | O auxiliar de decisão pede aos doentes que pensem sobre quais os aspetos positivos e negativos das opções são mais importantes para eles (de forma implícita ou explícita). | Claro Não Claro  Comentários: | 1 2 3 4  Comentários: |
| QU9 | O auxiliar de decisão inclui um método passo-a-passo para a tomada de decisão. | Claro Não Claro  Comentários: | 1 2 3 4  Comentários: |
| QU10 | O auxiliar de decisão inclui ferramentas como fichas de trabalho ou listas de perguntas para utilizar ao discutir as opções com um profissional de saúde. | Claro Não Claro  Comentários: | 1 2 3 4  Comentários: |
| QU11 | O processo de desenvolvimento incluiu uma avaliação de necessidades com os clientes ou doentes. | Claro Não Claro  Comentários: | 1 2 3 4  Comentários: |
| QU12 | O processo de desenvolvimento incluiu uma avaliação de necessidades com os profissionais de saúde. | Claro Não Claro  Comentários: | 1 2 3 4  Comentários: |
| QU13 | O processo de desenvolvimento incluiu a revisão por clientes/doentes não envolvidos na produção da intervenção de apoio à decisão. | Claro Não Claro  Comentários: | 1 2 3 4  Comentários: |
| QU14 | O processo de desenvolvimento incluiu a revisão por profissionais não envolvidos na produção da intervenção de apoio à decisão. | Claro Não Claro  Comentários: | 1 2 3 4  Comentários: |
| QU15 | O auxiliar de decisão foi testado na prática com doentes que tinham de tomar a decisão. | Claro Não Claro  Comentários: | 1 2 3 4  Comentários: |
| QU16 | O auxiliar de decisão foi testado na prática com profissionais de saúde que aconselham doentes que estão perante a decisão. | Claro Não Claro  Comentários: | 1 2 3 4  Comentários: |
| QU17 | O auxiliar de decisão (ou a documentação associada) descreve a forma como a evidência científica foi selecionada ou resumida. | Claro Não Claro  Comentários: | 1 2 3 4  Comentários: |
| QU18 | O auxiliar de decisão (ou a documentação associada) descreve a qualidade da evidência científica utilizada. | Claro Não Claro  Comentários: | 1 2 3 4  Comentários: |
| QU19 | O auxiliar de decisão inclui as credenciais ou habilitações dos autores/responsáveis pelo desenvolvimento da ferramenta. | Claro Não Claro  Comentários: | 1 2 3 4  Comentários: |
| QU20 | O auxiliar de decisão (ou a documentação associada) indica os níveis de legibilidade (utilizando 1 ou mais das escalas disponíveis). | Claro Não Claro  Comentários: | 1 2 3 4  Comentários: |
| QU21 | Existe evidência de que o auxiliar de decisão aumenta a correspondência entre as preferências do doente informado e a opção selecionada. | Claro Não Claro  Comentários: | 1 2 3 4  Comentários: |
| QU22 | Existe evidência de que o auxiliar de decisão ajuda a aumentar o conhecimento dos doentes sobre as características das opções existentes. | Claro Não Claro  Comentários: | 1 2 3 4  Comentários: |
| QU23 | O auxiliar de decisão inclui informações sobre a probabilidade de um teste ter um resultado verdadeiro positivo. | Claro Não Claro  Comentários: | 1 2 3 4  Comentários: |
| QU24 | O auxiliar de decisão inclui informações sobre a probabilidade de um teste ter um resultado verdadeiro negativo. | Claro Não Claro  Comentários: | 1 2 3 4  Comentários: |
| QU25 | O auxiliar de decisão inclui informações sobre a probabilidade de um teste ter um resultado falso positivo. | Claro Não Claro  Comentários: | 1 2 3 4  Comentários: |
| QU26 | O auxiliar de decisão inclui informações sobre a probabilidade de um teste ter um resultado falso negativo. | Claro Não Claro  Comentários: | 1 2 3 4  Comentários: |
| QU27 | O auxiliar de decisão descreve a probabilidade de a doença ser detetada com e sem a utilização do teste. | Claro Não Claro  Comentários: | 1 2 3 4  Comentários: |

**APPENDIX 3 – Synthesis I meeting comments.**

**Translation, Cultural Adaptation and Validation of the “IPDAS Minimal Criteria v.4.0 (2013) instrument” for the Portuguese language**

**Step 2: Synthesis I**

| T3 Preliminary Translation | Comments |
| --- | --- |
| Critérios de qualificação: critérios considerados de carácter de definição. Para serem consideradas para certificação e classificadas como auxiliar de decisão do doente, as ferramentas deverão satisfazer todos os critérios de qualificação. | It was decided to use the term “patient decision aid”. It was suggested by Prof. Stacey to use the abbreviation “decision aid” throughout the rest of the document. |
| Q1: O auxiliar à decisão do doente descreve a doença ou o problema de saúde (tratamento, procedimento ou exame) para o qual é necessária a decisão em causa. | Many doubts regarding the term “index decision”. It was decided to use the term “decision in question”. |
| Q2: O auxiliar à decisão do doente indica explicitamente a decisão que terá de ser considerada (decisão em causa). | Many doubts regarding the term “index decision”. It was decided to use the term “decision in question”. |
| Q3: O auxiliar à decisão do doente identifica o público-alvo. |  |
| Q4: O auxiliar à decisão do doente descreve as opções disponíveis para a decisão em causa. | Many doubts regarding the term “index decision”. It was decided to use the term “decision in question”. |
| Q5: O auxiliar à decisão do doente descreve as características positivas (benefícios ou vantagens) de cada opção. | “Characteristics” are changed to “aspects”, similar to what is used in C1 and QU2. |
| Q6: O auxiliar à decisão do doente descreve as características negativas (danos, efeitos indesejáveis ou desvantagens) de cada opção. | None of the forward translations chose “efeitos indesejáveis” as a translation of “side effects”. This is the correct term, although “efeitos secundários” is the term commonly used. We should also consider the use of “riscos” as a translation of “harm”.  The research team opted to maintain “efeitos indesejáveis” and “danos”.  “Characteristics” are changed to “aspects”, similar to what is used in C1 and QU2. |
| Q7: O auxiliar à decisão do doente descreve o impacto das consequências das opções (por exemplo, físicas, psicológicas, sociais) e/ou inclui um exercício de valorização que pede aos doentes para ponderarem ou classificarem os aspetos positivos ou negativos que são mais importantes para eles. | None of the forward translations chose “exercício de valorização” as a translation of “value clarification exercise”. In my interpretation, this is the intended translation.  “Explicit” has been added to clarify the type of valuation exercise that should be included in the decision aid. |
| Critérios de certificação: critérios que são essenciais para evitar riscos ou parcialidades prejudiciais. As ferramentas devem cumprir todos os critérios de certificação para cumprir as normas de certificação. | Clarification of the term “harmful bias”. Consensus translation: “risk of harmful bias”. The research team chose to rewrite as "the risk of a biased decision". |
| C1: O auxiliar à decisão do doente apresenta os aspetos positivos e negativos das opções de forma semelhante (por exemplo, usando tipos de letra iguais, mesma sequência, mesma apresentação de informações estatísticas). |  |
| C2: O auxiliar à decisão do doente (ou a documentação associada) inclui citações das evidências selecionadas. | The term is changed to “evidência”, since it is usually used in the singular. |
| C3: O auxiliar à decisão do doente (ou a documentação associada) inclui uma data de redação ou publicação. |  |
| C4: O auxiliar à decisão do doente (ou a documentação associada) inclui informações sobre a política de atualização. |  |
| C5: O auxiliar à decisão do doente inclui informações sobre os níveis de incerteza de um acontecimento ou probabilidade de determinados desfechos (por exemplo, indicando um intervalo ou utilizando afirmações como “na nossa melhor estimativa [...]”). | None of the forward translations chose “acontecimento” as a translation of “event”. This is the correct term, although I recognise that “evento” can also be used.  It was decided to use “níveis de incerteza quanto à probabilidade de um determinado evento ou desfecho” |
| C6: O auxiliar à decisão do doente (ou a documentação associada) inclui informações sobre o financiamento usado para desenvolver a ferramenta. |  |
| C7: O auxiliar à decisão do doente descreve aquilo que o teste se destina a avaliar. | None of the c opted for this translation. However, the present formulation is clearer. |
| C8: Se o teste se destina a detetar a doença ou o problema de saúde, o auxiliar à decisão do doente descreve os próximos passos habituais. | None of the forward translations opted for this translation. However, this formulation ensures consistency and clarity.  Changed to “Se o teste detetar”. |
| C9: O auxiliar à decisão do doente descreve os próximos passos, se a doença ou o problema de saúde não for detetado. | “de saúde” added to ensure consistency and clarity. |
| C10: O auxiliar à decisão do doente inclui informações sobre as consequências de detetar um problema de saúde ou doença que nunca teria causado problemas caso não tivesse sido feito um rastreio (viés de antecipação diagnóstica). |  |
| Critérios de qualidade: critérios que são desejáveis porque vão melhorar um auxiliar à decisão, mas que não são essenciais para reduzir o risco de parcialidades prejudiciais. Estes aspetos melhorariam a experiência de utilização do auxiliar à decisão, mas não seria expectável que a inexistência dos mesmos influenciasse a decisão de uma pessoa de forma negativa. | As discussed in the “Certification Criteria”, “the risk of a biased decision” will be used instead of “risk of harmful bias”. |
| QU1: O auxiliar à decisão do doente descreve o curso natural da doença ou problema de saúde se não for tomada qualquer medida (quando apropriado). | “tomar qualquer ação” is a transfer of the English “take any action”. Correct translation is “tomar qualquer medida”. |
| QU2: O auxiliar à decisão do doente possibilita a comparação entre os aspetos positivos e negativos das opções disponíveis. |  |
| QU3: O auxiliar à decisão do doente inclui informações sobre as probabilidades de desfecho associadas às opções (ou seja, as consequências prováveis das decisões). |  |
| QU4: O auxiliar à decisão do doente especifica as taxas de acontecimentos para as probabilidades de desfecho. | “occurrence rates” could be a possible translation for “event rates”  It was decided to use “event occurrence rates for outcome probabilities” (“taxas de ocorrência do evento para as probabilidades do desfecho”). |
| QU5: O auxiliar à decisão do doente permite ao utilizador comparar as probabilidades de desfecho de várias opções no mesmo período de tempo (quando possível). |  |
| QU6: O auxiliar à decisão do doente permite ao utilizador comparar as probabilidades de desfecho de várias opções usando o mesmo denominador (quando possível). |  |
| QU7: O auxiliar à decisão do doente possibilita visualizar as probabilidades de mais do que uma forma (por exemplo, em texto, números e diagramas). |  |
| QU8: O auxiliar à decisão do doente pede aos doentes que pensem sobre quais os aspetos positivos e negativos das opções são mais importantes para eles (de forma implícita ou explícita). |  |
| QU9: O auxiliar à decisão do doente inclui um método passo-a-passo para a tomada de decisão. |  |
| QU10: O auxiliar à decisão do doente inclui ferramentas como fichas de trabalho ou listas de perguntas para utilizar ao discutir as opções com um profissional de saúde. | Worksheet translation was discussed. Consensus on the use of “fichas de trabalho”. |
| QU11: O processo de desenvolvimento incluiu uma avaliação de necessidades com os clientes ou doentes. |  |
| QU12: O processo de desenvolvimento incluiu uma avaliação de necessidades com os profissionais de saúde. |  |
| QU13: O processo de desenvolvimento incluiu a revisão por clientes/doentes não envolvidos na produção da intervenção de apoio à decisão. |  |
| QU14: O processo de desenvolvimento incluiu a revisão por profissionais de saúde não envolvidos na produção da intervenção de apoio à decisão. | It was decided to withdraw “de saúde” (it may not be developed by a health professional). |
| QU15: O auxiliar à decisão do doente foi testado na prática com doentes que tinham de tomar a decisão. |  |
| QU16: O auxiliar à decisão do doente foi testado na prática com profissionais de saúde que tinham de tomar a decisão. | It is not the health professionals who make the decision, but those who advise the patient who will make the decision. Thus, it changed to “profissionais de saúde que aconselham doentes que estão perante a decisão”. |
| QU17: O auxiliar à decisão do doente (ou a documentação associada) descreve a forma como as evidências científicas foram selecionadas ou resumidas. | The term is changed to “evidência”, since it is usually used in the singular. |
| QU18: O auxiliar à decisão do doente (ou a documentação associada) descreve a qualidade das evidências científicas utilizadas. | The term is changed to “evidência”, since it is usually used in the singular. |
| QU19: O auxiliar à decisão do doente inclui as credenciais ou habilitações dos autores/responsáveis pelo desenvolvimento da ferramenta. | “qualificações” is a transfer from English. The correct term is “habilitações”. |
| QU20: O auxiliar à decisão do doente (ou a documentação associada) indica os níveis de legibilidade (utilizando 1 ou mais das escalas disponíveis). | Readability: Quality of what is readable ("legibility", in Dicionário Priberam da Língua Portuguesa [online], 2008-2023, https://dicionario.priberam.org/legabilidade).  Intelligibility: Quality of what can be understood, of what is intelligible ("intelligibility", in Dicionário Priberam da Língua Portuguesa [online], 2008-2023, https://dicionario.priberam.org/inteligabilidade).  Given that English refers to “readability” (and not “comprehensibility”), the term readability seems more adequate.  This issue was clarified with Prof. Stacey, who confirmed that the term “readability” relates to “legibilidade”. |
| QU21: Existem evidências de que o auxiliar à decisão do doente aumenta a correspondência entre as preferências do doente informado e a opção selecionada. | The term is changed to “evidência”, since it is usually used in the singular. |
| QU22: Existem evidências de que o auxiliar à decisão do doente ajuda a aumentar o conhecimento dos doentes sobre as características das opções existentes. | The term is changed to “evidência”, since it is usually used in the singular. |
| QU23: O auxiliar à decisão do doente inclui informações sobre a probabilidade de um teste ter um resultado verdadeiro positivo. |  |
| QU24: O auxiliar à decisão do doente inclui informações sobre a probabilidade de um teste ter um resultado verdadeiro negativo. |  |
| QU25: O auxiliar à decisão do doente inclui informações sobre a probabilidade de um teste ter um resultado falso positivo. |  |
| QU26: O auxiliar à decisão do doente inclui informações sobre a probabilidade de um teste ter um resultado falso negativo. |  |
| QU27: O auxiliar à decisão do doente descreve a probabilidade de a doença ser detetada com e sem a utilização do teste. |  |
